# Ångström-resolution imaging of cell-surface glycans

**DOI:** 10.1038/s41565-025-01966-5

**Published:** 2025-07-28

**Authors:** Luciano A. Masullo, Karim Almahayni, Isabelle Pachmayr, Monique Honsa, Larissa Heinze, Sarah Fritsche, Heinrich Grabmayr, Ralf Jungmann, Leonhard Möckl

**Affiliations:** 1 Max Planck Institute of Biochemistry, Planegg, Germany; 2 Max Planck Institute for the Science of Light, Erlangen, Germany; 3 Department of Physics, Friedrich-Alexander-University Erlangen-Nuremberg, Erlangen, Germany; 4 Faculty of Physics and Center for Nanoscience, Ludwig Maximilian University, Munich, Germany; 5 Faculty of Medicine/CITABLE, Friedrich-Alexander-University Erlangen-Nuremberg, Erlangen, Germany; 6 Deutsches Zentrum Immuntherapie, University Clinic Erlangen, Erlangen, Germany

**Keywords:** Nanophotonics and plasmonics, Nanobiotechnology, Super-resolution microscopy

## Abstract

Glycobiology is rooted in the study of monosaccharides, ångström-sized molecules that are the building blocks of glycosylation. Glycosylated biomolecules form the glycocalyx, a dense coat encasing every human cell with central relevance—among others—in immunology, oncology and virology. To understand glycosylation function, visualizing its molecular structure is fundamental. However, the ability to visualize the molecular architecture of the glycocalyx has remained challenging. Techniques such as mass spectrometry, electron microscopy and fluorescence microscopy lack the necessary cellular context, specificity and resolution. Here we combine resolution enhancement by sequential imaging with metabolic labelling, enabling the visualization of individual sugars within glycans on the cell surface, thus obtaining images of the glycocalyx with a spatial resolution down to 9 Å in an optical microscope.

## Abstract (web summary)

By combining bioorthogonal metabolic labelling and resolution enhancement through sequential imaging of DNA barcodes, the molecular organization of individual sugars in the native glycocalyx has been resolved at a spatial resolution of 9 ångström.

## Main

The glycocalyx covers all cells in the human body. It is composed of glycosylated proteins, glycolipids, free polysaccharides and the recently discovered glycoRNAs^1,2^. The glycocalyx has a fundamental role in a range of cellular processes in health and disease, including immune system regulation^3^, cell signalling^4,5^, leukocyte adhesion^6^ and cancer development^7^. In particular, glycans within the glycocalyx mediate critical interactions with the microenvironment—including microbiota and immune cells—shaping immune responses, microbial colonization and cellular communication^8,9^. Dissecting the spatial organization of the glycocalyx is therefore crucial to uncover how alterations in glycan distribution contribute to disease onset and progression^10^.

A critical challenge in glycobiology has been the structural analysis of cell-surface glycans, which are the fundamental components of the glycocalyx at the molecular level. This question has been addressed by a number of advanced characterization methods, in particular, mass spectrometry^11^, scanning tunnelling microscopy^12^, electron microscopy^13^ and light microscopy^14^. Each method revealed important aspects of cell-surface glycosylation. Mass spectrometry-based methods were used to infer glycan structures^15^; scanning tunnelling microscopy allowed for the visualization of isolated free glycans and glycans attached to proteins and lipids^16^, and light as well as electron microscopy enabled measurements of glycocalyx thickness in tissue sections and cultured cells^14,17^.

However, each of these methods has limitations. Mass spectrometry requires the removal of glycans from the cell surface for ionization^11^; scanning tunnelling microscopy also requires the isolation of glycans^16^; electron microscopy sample preparation is damaging to the glycocalyx and lacks species-specific contrast^18^. Fluorescence microscopy would be an ideal method to study the glycocalyx in situ owing to its low invasiveness and cellular compatibility. To spatially analyse glycocalyx structure with molecular specificity in cells using fluorescence microscopy, one key ingredient is efficient and specific tagging of target sugars. However, the field of glycobiology has traditionally suffered from the limited availability of methods to specifically and efficiently label structural building units, compared with DNA, RNA or protein biology. In particular, there is a limited availability of antibodies against glycan structures, which, in addition, often exhibit low affinity^19^. Furthermore, genetic labelling approaches are not applicable, as glycans are secondary gene products and therefore not directly encoded in the genome^20^. Finally, lectin-based glycan labelling can be used; however, lectins show rather poor affinity and specificity^21^.

Recently, the discovery of metabolic incorporation of unnatural sugar analogues in conjunction with live-cell click chemistry has provided the field of functional glycobiology with an asset to specifically label individual sugar residues within the glycocalyx. This ability has been used for fluorescence microscopy^22^ and even super-resolution approaches^14,23^. However, the expected distances between individual sugars (often densely packed within the glycocalyx) based on available structural studies^24,25^ are at the sub-10 nm scale and even below 1 nm. While in principle click chemistry holds the potential to achieve sub-nm resolution owing to the size of the labelling molecule (8 Å), conventional super-resolution microscopy methods such as STORM (stochastic optical reconstruction microscopy)^26^ lack the spatial resolution to resolve the molecular architecture of the glycocalyx^27^. While previous studies enabled relevant insights^14,28^, they still essentially lacked the resolution to resolve details below 20 nm. Moreover, recent reports^29^ indicate that, at distances below 10 nm, photophysical interactions between fluorophores substantially influence fluorescence emission, thereby limiting super-resolution techniques that rely on labelling the imaging target with a single, fixed dye to resolutions of approximately 10–20 nm. A method to study cell-surface glycans at molecular resolution within the native cellular context is missing so far.

We have recently introduced RESI (resolution enhancement by sequential imaging)^30^, a DNA-barcoding optical microscopy method that achieves ångström resolution. While ångström resolution was demonstrated in DNA origami nanostructures, the achievable resolution in cells was experimentally limited to the size of the labelling probes, that is, nanobodies of around 5 nm in size. Thus, achieving ångström resolution in cells has remained elusive and limited by the labelling approach.

Here we combine RESI^30^ with bioorthogonal metabolic labelling^31,32^, which allows us to resolve glycans down to individual sugars with ångström resolution in whole cells. Leveraging this unique spatial information, we show that sugar residues form distinct spatial arrangements on the surface of cells that are smaller than the size of single proteins. In-depth quantitative analysis reveals previously elusive molecular signatures of single sugars within glycans on individual proteins. Taken together, we establish RESI combined with metabolic labelling as a transformative technique in glycobiology with the prospect of linking glycan structure to function, identifying molecular glycocalyx changes related to disease progression, discovering novel therapeutic avenues and developing diagnostic tools. From a methodological perspective, our work constitutes the first demonstration of optical ångström resolution in a native cellular context.

## Metabolic labelling of sugars with DNA barcodes

**Fig. 1:**
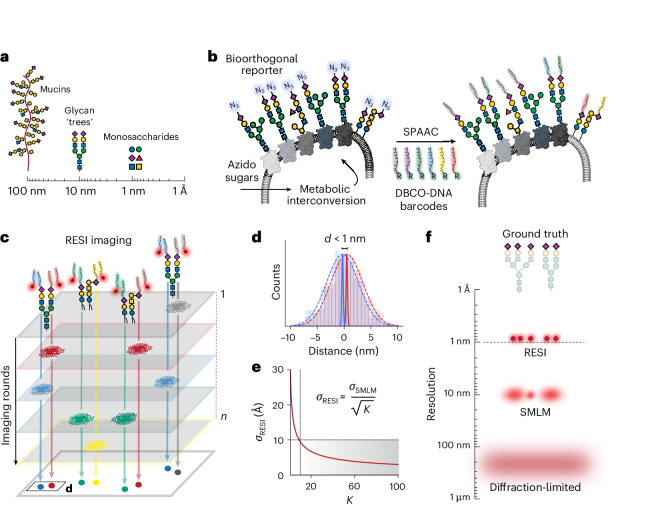
Experimental concept. **a**, Monosaccharides represent the smallest length scale in glycobiology, forming the fundamental building blocks for larger glycan structures (tens of nanometres) and heavily glycosylated mucins (up to hundreds of nanometres). Monosaccharides are depicted following the Symbol Nomenclature for Glycans (SNFG) guidelines^53^. **b**, Azido sugars are metabolized by cells and integrated into target monosaccharides introducing a bioorthogonal azido group as a molecular reporter. This azido group facilitates the attachment of six orthogonal DBCO-modified DNA strands (shown in different colours) via a strain-promoted azide-alkyne click chemistry reaction (SPAAC), enabling precise labelling of the target monosaccharide unit. **c**, Imaging sugar molecules, labelled with distinct DNA barcodes, through the sequential addition of their corresponding imaging DNA sequences, facilitates temporal separation of signals distinguishing blinks from nearby molecules. **d**, Combining all localizations per target (*K*) from each imaging round enhances localization precision. **e**, In RESI, localization precision improves with $1/\sqrt{K}$; thus, resolution enhancement is independent of the localization precision of single fluorescent molecules (*σ*_SMLM_) and ångström-scale precision can be achieved. **f**, Unlike other super-resolution imaging techniques, RESI is capable of resolving single sugars within a glycan. The glycan structures exemplify sialic acid labelling.

Monosaccharides, just a few ångströms in size, serve as the building blocks of complex glycans and glycosylated biomolecules (Fig. 1a). To resolve these cell-surface carbohydrates, we combine efficient and specific metabolic labelling with the ångström spatial resolution of RESI. First, we developed and optimized a method to attach single strands of DNA to the sugars of interest (Fig. 1b). RESI is based on DNA-PAINT (DNA points accumulation for imaging in nanoscale topography)^33^, a super-resolution microscopy method that relies on the transient binding of fluorescently labelled DNA probes to complementary target sequences, achieving approximately 5–10 nm spatial resolution through single-molecule localization. By stochastically isolating and sequentially imaging sparse subsets of targets at this resolution (Fig. 1c), RESI increases the precision of DNA-PAINT measurements by averaging localizations (Fig. 1d), enabling spatial resolution at the ångström level (Fig. 1e). This is critical to resolve individual glycans and individual sugars within glycans (Fig. 1f).

**Extended Data Fig. 1:**
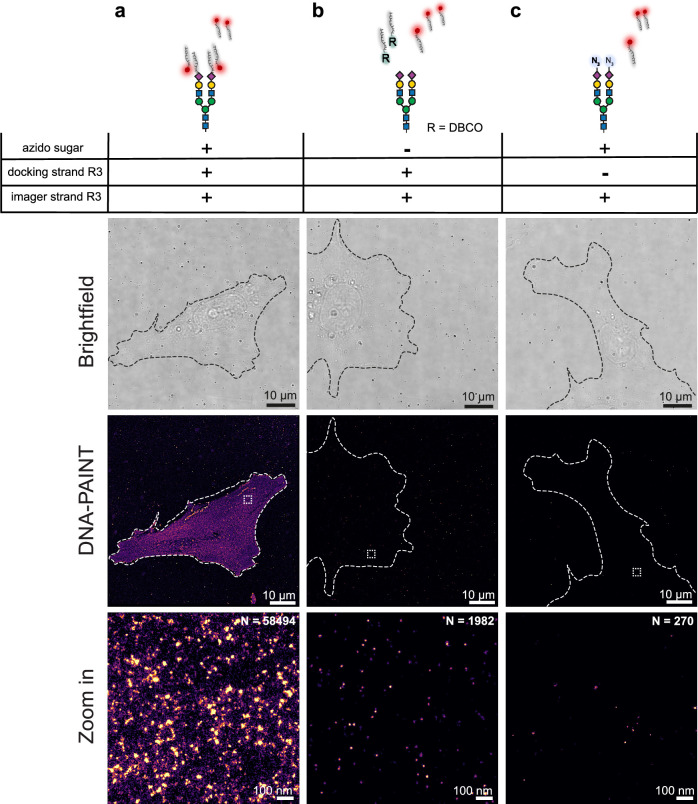
Validation of the labeling workflow. **a**, Specificity of sialic acid labeling is demonstrated in comparison to controls: **b**, samples prepared without azido sugars and **c**, samples prepared without DNA docking strands. The number of localizations (N) is indicated in the upper-right corner of the zoomed-in panels, highlighting the labeling specificity.

**Extended Data Fig. 2:**
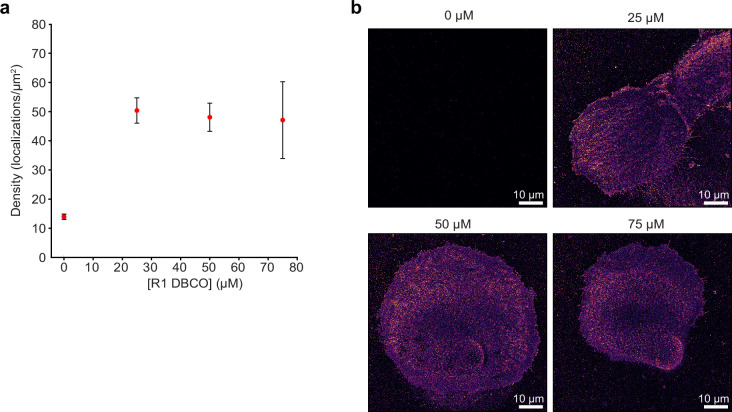
Saturation of azido binding sites. **a**, Graphical representation of binding site saturation as a function of DBCO-R1 concentration. **b**, Representative images demonstrating binding site saturation. Error bars indicate the standard error of the mean (SEM). Statistics derived from n = 6 different HMECs labeled with ManNAz (technical replicate). Source data

To enable RESI imaging of individual sugar residues within glycans, we optimized metabolic labelling to facilitate efficient attachment of DNA probes. In particular, we used tetraacetylated *N*-acetylgalactosamine (Ac_4_GalNAz) to label *N*-acetyllactosamine (LacNAc; that is, Galβ1-4GlcNAc) residues and tetraacetylated *N*-acetylmannosamine (Ac_4_ManNAz) to label *N*-acetylneuraminic acid (Neu5Ac) with azido sugars. In the following, Neu5Ac is referred to as ‘sialic acids’ for simplicity. The azido sugars are subsequently covalently linked to six orthogonal dibenzocyclooctyne (DBCO)-modified RESI-compatible DNA strands via strain-promoted live-cell copper-free click chemistry^32^ (Extended Data Fig. 1). We opted for copper-free click chemistry, as it has been shown to achieve higher labelling efficiencies compared with copper-catalysed click chemistry^27^. The concentration of docking strands was optimized to ensure complete saturation of available azido groups (Extended Data Fig. 2).

In the context of RESI’s ångström-resolution capabilities, metabolic labelling offers the critical advantage of a much smaller labelling footprint (below 1 nm) compared with antibody (10–15 nm) and even nanobody-based labelling (5–10 nm) as the DNA strands are attached directly to the sugar target without the need for a genetically encoded tag or exogenous affinity reagents.

## Visualization of glycans in situ with ångström resolution

**Fig. 2:**
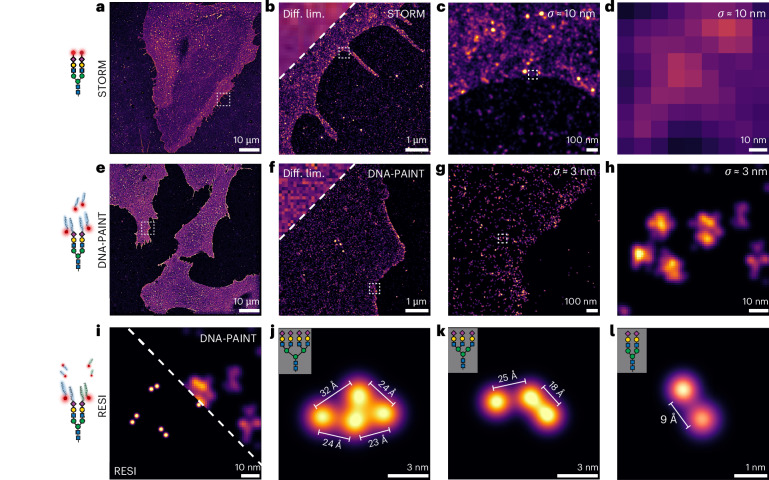
Visualization of cell-surface sialic acids with ångström resolution. **a**, Overview (d)STORM image of sialic acids on HMECs treated with ManNAz and labelled with A647-DBCO. **b**, A side-by-side comparison of the diffraction-limited (Diff. lim.; top left corner) and (d)STORM images shows improvement in resolution. **c**, Zoomed-in (d)STORM image showing sialic acids on the cell surface. **d**, Close-up view illustrating the limitations of (d)STORM in resolving individual sugar molecules. **e**, Overview DNA-PAINT image of sialic acids on HMECs treated with ManNAz and labelled with a single DBCO-modified DNA sequence. **f**, A side-by-side comparison of diffraction-limited (top left corner) and DNA-PAINT images shows improved resolution. **g**, Zoomed-in DNA-PAINT image showing sialic acids on the cell surface. **h**, A close-up DNA-PAINT image showing approximately threefold improvement in resolution compared with (d)STORM, although individual glycans remain unresolved. **i**, Side-by-side comparison of RESI (left) and DNA-PAINT (right) capabilities in resolving sialic acids. Only RESI, but not DNA-PAINT, allows the detection of sub-10 nm sugar–sugar distances. **j**, Tetra-antennary glycan on the cell surface. **k**, Tri-antennary glycan on the cell surface. **l**, Bi-antennary glycan capped with sialic acid residues that are 9 Å apart.

To demonstrate the capability of our approach, we imaged human microvascular endothelial cells (HMECs) where sialic acids were tagged with azido groups via Ac_4_ManNAz incorporation in order to benchmark spatial resolution using total internal reflection fluorescence (TIRF), STORM, DNA-PAINT and RESI. First, we used cells labelled with DBCO-Alexa Fluor 647 and imaged them using diffraction-limited TIRF microscopy^34^ at approximately 250 nm resolution. The same sample was then imaged with STORM^26,35^ achieving around 10 nm localization precision (25 nm resolution) (Fig. 2a–d). Despite offering spatial resolution an order of magnitude better than the diffraction limit (Fig. 2b), STORM fails to resolve the details of the sugar distribution at the true molecular scale (Fig. 2d).

We then proceeded to HMECs labelled with DBCO-ssDNA using six different DNA sequences (Methods). Figure 2e shows a DNA-PAINT image encompassing several HMECs within a 100 × 100 μm^2^ field of view (FOV). Figure 2f–h shows successive zoom-ins. At approximately 3 nm localization precision (7 nm resolution), DNA-PAINT offers approximately a threefold resolution increase compared with STORM (Fig. 2f) but still fails to resolve individual glycans (Fig. 2h), not to mention individual sugars within glycans. Only RESI (Fig. 2i–l) at up to 3 Å localization precision allows us to resolve the molecular details of the glycocalyx, unveiling the spatial distribution and structure of single glycans and most excitingly their constituent sugars. For example, we resolve isolated clusters of single sugars that are compatible with bi-, tri- and tetra-antennary glycans (Fig. 2j–l). Notably, as shown in Fig. 2l, we can resolve distances down to 9 Å between two single sugar residues in a glycan.

This demonstrates optical ångström resolution in the native cellular context, extending fluorescence microscopy more than 250-fold over the diffraction limit of light while maintaining its main advantages in terms of low invasiveness and molecular specificity. Our approach combining RESI and metabolic labelling allows us to characterize the spatial arrangement of individual sugar residues within the native glycocalyx at previously unattainable molecular resolution. This achievement also represents the first demonstration of ångström-resolution imaging of a complex, spatially extended structure in a whole cell, advancing beyond in vitro demonstrations^30,36^.

## Molecular arrangement of sugars on the cell surface

**Fig. 3:**
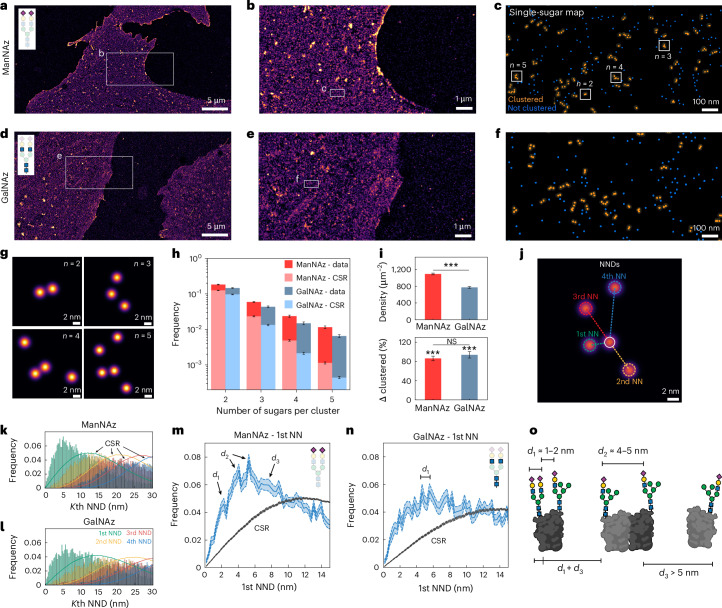
Identification of distinct nanoscale glycan fingerprints. **a**, HMECs treated with ManNAz, targeting sialic acids, and labelled with six orthogonal DBCO-modified DNA sequences. **b**, RESI zoom-in showing sialic acid residues on the cell surface. **c**, Single sugars are clustered using DBSCAN. **d**, HMECs treated with GalNAz, targeting LacNAc residues, and labelled with six orthogonal DBCO-modified DNA sequences**. e**, RESI zoom-in showing LacNAc residues on the cell surface. **f**, Representative clusters of LacNAc residues. **g**, Single sugars visualized within individual clusters. **h**, Frequency of clusters with five or fewer sugars compared with CSR. Height of the bar plot represents the mean value and error bar represents the standard error of the mean. **i**, Sugar density on the cell surface (top) and relative variation of the amount of clustered sugars (Δ clustered) compared with CSR (bottom). Height of the bar plot represents the mean value and error bar represents the standard error of the mean. Statistics derived from *n* = 40 different areas picked from 6 different cells (technical replicate) for ManNAz and *n* = 40 different areas picked from 6 different cells (technical replicate) for GalNAz. A one-sided Student *t*-test was used to compare Δ clustered to the null hypothesis (*P* = 4.3 × 10^−17^ (ManNAz) and *P* = 8.9 × 10^−21^ (GalNAz)) and a two-sided Student *t*-test was used to compare Δ clustered between ManNAz and GalNAz (*P* = 0.34). ***P < 0.001. NS, not significant (*P* > 0.05). **j**, Visual representation of first (green), second (yellow), third (red) and fourth (blue) nearest sugar distances for a given localization (white). **k**, First to fourth nearest-neighbour sialic acid distances (histograms) compared with CSR (dashed line). First, second, third and fourth NNDs are shown in green, yellow, red and blue, respectively. **l**, First to fourth nearest-neighbour LacNAc distances (histograms) compared with CSR (solid line). First, second, third and fourth NNDs are shown in green, yellow, red and blue, respectively. **m**, First NNDs for sialic acids (blue) and CSR (grey). The solid line represents the mean value and the dotted lines and the shaded area represent the confidence interval of 67% (1 s.d.). **n**, First NNDs for LacNAc residues (blue) and CSR (grey). The solid line represents the mean value and the dotted lines and the shaded area represent the confidence interval of 67% (1 s.d.). **o**, The quantitative analysis of the data is compatible with sugars located on different glycans of the same protein and sugars on neighbouring glycoproteins. Source data

**Extended Data Fig. 3:**
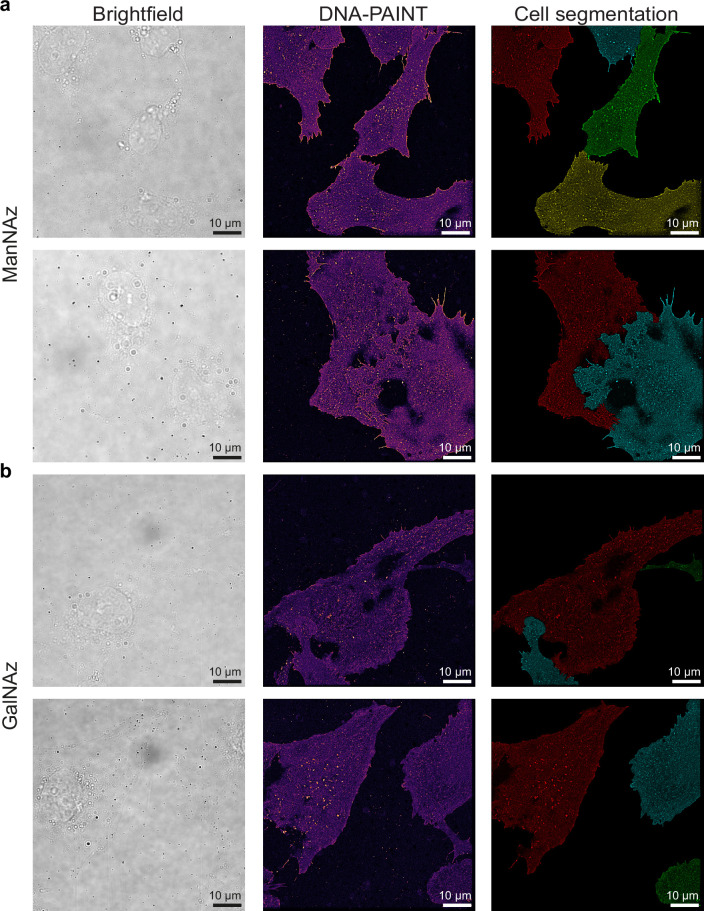
Brightfield images and cell segmentation. **a**, HMECs labeled with ManNAz. **b**, HMECs labeled with GalNAz. The left panels show brightfield images, the middle panels display DNA-PAINT overviews, and the right panels show segmented cells, with each cell depicted in a different color.

Subsequently, we focused on extracting detailed quantitative information from the single-sugar-resolved datasets. We imaged HMECs labelled with Ac_4_ManNAz (Fig. 3a–c and Extended Data Fig. 3) and Ac_4_GalNAz (Fig. 3d–f and Extended Data Fig. 3), respectively. Figure 3a,d shows an overview of the cells, while Fig. 3b,e and Fig. 3c,f show successive zoom-ins down to the scale of individual sugar molecules.

To analyse the spatial distribution and molecular-scale organization of glycans on the cell surface, we applied the density-based spatial clustering of applications with noise (DBSCAN) clustering algorithm^37^ using a conservative distance threshold of *d* = 10 nm (Methods). This threshold was chosen based on an estimated typical glycan size of approximately 5–10 nm (ref. ^24^). By setting this value, we aimed to distinguish direct interactions (that is, sugars within the same glycan, occurring at distances <10 nm) from non-direct interactions (that is, sugars located on different glycans, occurring at distances >10 nm). Figure 3c,f shows clusters of sugars (orange) and single, non-clustered sugars (blue). Clusters of up to five sugars are detected on the cell surface (Fig. 3g). Canonical glycan branching stops at *n* = 4; however, a higher number of branches has been reported^38,39^. Thus, these clusters likely correspond to a single glycan with up to five branches or to more than one glycan bound to a protein.

To quantify the overall clusterization of sugar positions, we picked *N* = 40 different areas of 0.5 μm^2^ with apparently homogeneous density across different cells and different FOVs. We then histogrammed the number of sugars per cluster and compared the clusterization of the data with a complete spatial randomness (CSR) simulation at the same densities (Fig. 3h). Both Ac_4_ManNAz (red) and Ac_4_GalNAz (blue) show a significantly higher clusterization than the one expected for CSR. Interestingly, despite observing differences in the overall sugar densities, the relative clusterization increase from CSR is the same for both Ac_4_ManNAz and Ac_4_GalNAz (Fig. 3i), indicating that the detected clusterization is not a density-dependent effect. For both Ac_4_ManNAz- and Ac_4_GalNAz-mediated labelling, we observe average densities of approximately 1,100 and 750 sugars per µm^2^, respectively, indicating high unnatural sugar incorporation and DBCO-click labelling efficiency (Fig. 3i). These high densities are consistent with glycosylation being the most abundant post-translational modification^40^ and sialic acids serving as the most abundant terminal sugar on human cell-surface glycans^41^. Ac_4_ManNAz is processed via the sialic acid biosynthetic pathway, where it is converted into CMP-Neu5Az and incorporated at glycan termini. Ac_4_GalNAz enters the hexosamine biosynthetic pathway, where it is metabolized into UDP-GalNAz and UDP-GlcNAz and incorporated into the core structures of glycans, likely causing broader metabolization and lower cell-surface density compared with Ac_4_ManNAz.

**Extended Data Fig. 4:**
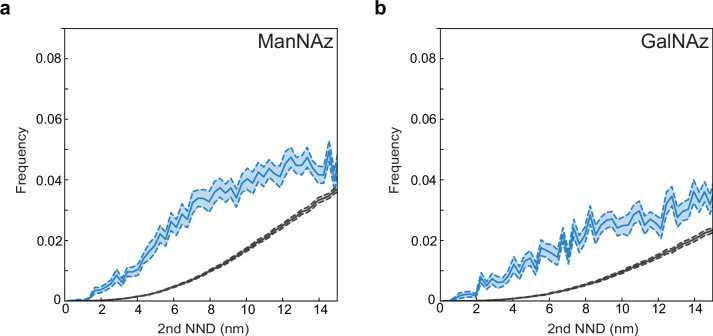
Second-nearest neighbor distances. **a**, HMECs labeled with ManNAz. Second-nearest neighbor distances for sialic acids (blue) and CSR (grey). The solid line represents the mean value and the dotted lines and the shaded area represent the confidence interval of 67% (1 SD). **b**, HMECs labeled with GalNAz. Second-nearest neighbor distances for LacNAc residues (blue) and CSR (grey). The solid line represents the mean value and the dotted lines and the shaded area represent the confidence interval of 67% (1 SD). Source data

The cell surface is a densely packed environment, where proteins and sugar molecules are tightly arrayed^42^. A single glycosylated protein, for example, can carry anywhere from a single glycan chain (for example, a glycosylated membrane protein) to hundreds of glycan chains (in the case of mucins)^43^. To further investigate the spatial arrangements within single-glycan length scales (up to 10 nm), we applied a nearest-neighbour distance (NND) analysis (Fig. 3j). In brief, the distances to the *K*th NN were calculated for each sugar up to *K* = 4 and the distances were then histogrammed to obtain the NND distributions for both ManNAz (Fig. 3k and Extended Data Fig. 4) and GalNAz (Fig. 3l and Extended Data Fig. 4). All *K*th NND distributions show a clear deviation from the CSR simulations (solid lines) at the same densities, indicating distinct molecular patterns at the sub-10 nm scale.

**Extended Data Fig. 5:**
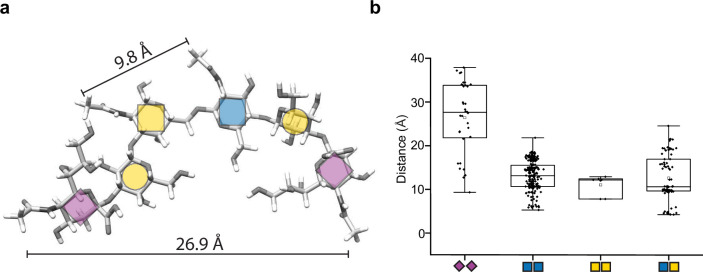
Sugar-sugar distances within a glycan. **a**, Theoretical sugar-sugar distances measured within a glycan. **b**, Sugar-sugar distances between sialic acids (labeled with ManNAz) and GlcNAc/GalNAc (labeled with GalNAz) measured across glycan structures listed on GlycoShape^65^. The centre line represents the median (50th percentile), the bounds of the box indicate the first (25th percentile) and third quartiles (75th percentile), the whiskers extend to the minimum and maximum values. Regarding power, 38 sia-sia distances from 17 glycans, 237 Glc-Glc distances from 52 glycans, 9 Gal-Gal distances from 9 glycans, 88 Glc-Gal distances from 44 glycans. Source data

A closer inspection of the 1st NND distributions shows distinct peaks at approximately 2 nm, 4 nm and 6 nm for sialic acids (Fig. 3m) and sub-6 nm peaks spaced by approximately 1 nm for Ac_4_GalNAz-labelled cells (Fig. 3n). Given that the average diameter of an integral membrane protein is approximately 5 nm (ref. ^44^), the distances of ManNAz are fully consistent with (i) sialic acids capping the same glycan (Extended Data Fig. 5), (ii) sialic acids located on different glycans of the same protein and (iii) sialic acids on neighbouring glycoproteins, respectively (Fig. 3o). However, while sialic acids are located at the terminal positions of glycan chains (Fig. 3m), LacNAc residues are more densely packed and positioned closer together owing to their presence within the glycan core (Fig. 3n). Here distinct peaks at distances of approximately 1 nm, 2 nm and 3 nm are visible. These are consistent with in silico distance measurements, where a set of biologically relevant glycan structures was surveyed (Extended Data Fig. 5).

## Conclusion

Our work provides the first visualization of the molecular structure of the glycocalyx in its native cellular context. This capability is poised to have far-reaching implications for functional glycobiology and cell biology in general. Glycocalyx components can now be studied in their native environment with spatial resolution down to a single sugar unit, which has been considered one of the central desiderata of glycobiology^45^.

A persistent challenge in glycobiology has been the lack of a defined composition for a healthy glycocalyx: its aberrant states still rely on the same fundamental building blocks—there are no inherently ‘diseased’ glycocalyx components per se. This complicates drug discovery in this field, as both excessive and insufficient levels of certain glycocalyx constituents can lead to severe side effects^46^. For example, hypersialylation can fuel tumour growth and enable immune evasion^47^, while hyposialylation may trigger autoimmune diseases^48^. By enabling ångström-resolution mapping of glycans, RESI could reveal what a healthy glycocalyx looks like at the molecular level and observe how drugs alter its organization. This opens the door to the rational development of highly specific glycocalyx-targeting therapies, transforming how we approach drug discovery in this field.

Understanding glycan-dependent processes such as glycan–receptor interactions requires imaging methods that can reveal glycans and interacting proteins with molecular resolution in a multiplexed fashion. By combining ångström-resolution imaging of glycans with expanded DNA barcodes^49^, one could simultaneously visualize membrane receptor proteins, providing new insights into their spatial organization and interactions. RESI is particularly well suited for multiplexing as it decouples species identification from fluorophore wavelength, instead encoding molecular identity in DNA sequences used for imaging^50^.

Furthermore, multi-target RESI of two monosaccharides could be achieved by introducing orthogonal functional groups on distinct sugars, such as periodate-mediated oxidation of sialic acids to generate aldehyde groups, combined with the metabolic incorporation of azido groups via *N*-azidoacetylgalactosamine (GalNAz)^14^. This strategy, along with the expanded DNA barcodes^49^, would enable multiplexed labelling and visualization of two monosaccharides within the same glycan structure, offering a more comprehensive understanding of the complexity and organization of the glycocalyx.

From an imaging technology point of view, we have shown that combining RESI with metabolic labelling unlocks the full potential of ångström-resolution fluorescence microscopy, demonstrating a resolution below 1 nm in a native cellular environment while maintaining FOVs of 100 × 100 μm^2^ comprising several cells per image acquisition. We envision that click chemistry approaches using unnatural amino acids^51^ could extend the ångström resolution achieved in glycans to proteins and other types of biomolecules.

From a methodological perspective, our work underscores the urgent need for rapid and extensive advancements in labelling glycocalyx constituents. While the frontier in glycocalyx research was once defined by the limitations of optical resolution, we have now surpassed those constraints, achieving single-sugar resolutions. However, the field continues to face an important gap in tools capable of effectively targeting and labelling relevant glycocalyx components.

While metabolic incorporation and bioorthogonal click chemistry represent the current state of the art for specific monosaccharides labelling, it is well recognized that neither approach achieves 100% efficiency. In our case, we observe a high density of resolved sugar residues (thousands of sugars per μm^2^); however, absolute quantification remains challenging. Crucially, our method shifts the limiting factor in glycocalyx analysis from optical resolution to labelling strategies. We anticipate that combining RESI with complementary analytical techniques such as liquid chromatography and mass spectrometry will enable direct quantification of labelling efficiency, providing a powerful platform to further refine glycan labelling and advance the field.

Looking ahead, expanding the current repertoire of metabolic labelling tools beyond those targeting LacNAc and sialic acids will be crucial, alongside driving the development of novel strategies for glycan labelling. RESI could substantially accelerate these efforts by enabling the precise analysis of labelling performance and providing insights into how incorporation efficiency is influenced by changes in the biosynthetic machinery of cells. With advancements in glycan labelling techniques, RESI is poised to resolve complete glycan structures, potentially enabling glycoproteomics using light microscopy.

Finally, given the growing importance of cancer glycoimmunology and clinical glycobiology, our approach could considerably deepen our understanding of the functional role of glycosylation in cancer biology. Precise mapping of changes in glycan organization, branching and density during cancer progression could add a new axis to the clinical analysis of glycosylation. Accordingly, our method could enable a completely new understanding of the role of cancer glycosylation in immune system regulation and immune evasion^52^. This will be of paramount importance for the identification of new diagnostic markers and targets in cancer immunotherapy, where glycosylation is already recognized as a central regulator. In conclusion, our findings will not only elucidate unknown areas of fundamental glycoscience but could also directly improve diagnosis and therapy.

## Methods

### Cell culture

All cells were cultured in T75 flasks (Corning BV) in a humidified atmosphere at 37 °C with 5% CO_2_. HMECs were cultured in MDCB-131 medium with 1% Glutamax, 10% fetal bovine serum, 10 ng ml^−1^ hEGF (all Thermo Fisher Scientific), 1 μg ml^−1^ hydrocortisone, and 1% of a penicillin–streptomycin solution containing 10,000 U ml^−1^ penicillin and 10 mg l^−1^ streptomycin (both Sigma-Aldrich). For imaging, cells were seeded on a Lab-Tek II Chambered Coverglass (Thermo Fisher Scientific).

### DNA-PAINT sequences

Six orthogonal DNA sequences modified with aza-dibenzocyclooctyne (DBCO) at their 5′ ends were used to label the azido sugars. The docking strand sequences used were 5xR1 (TCCTCCTCCTCCTCCTCCT), 5xR2 (ACCACCACCACCACCACCA), 7xR3 (CTCTCTCTCTCTCTCTCTC), 7xR4 (ACACACACACACACACACA), 5xR5 (CTTCTTCTTCTTCTTCTTC) and 5xR6 (AACAACAACAACAACAACAA). Their respective imagers were R1 (AGGAGGA-Cy3B), R2 (GGTGGT-Cy3B), R3 (GAGAGAG-Cy3B), R4 (TGTGTGT-Cy3B), R5 (GAAGAAG-Cy3B) and R6 (TG TTG TT-Cy3B). All sequences were speed-optimized^54^. See also Extended Data Table 2. The docking strand and imager sequences were purchased from Metabion.

### PCA, PCD and Trolox

Trolox (100×) was made by the addition of 100 mg of Trolox (Trolox, number 238813-5G, Sigma-Aldrich) to 430 μl of 100% methanol and 345 μl of 1 M NaOH in 3.2 ml of water. PCA (40×) was made by mixing 154 mg of PCA (PCA, number 37580-25G-F, Sigma-Aldrich) in 10 ml of water and NaOH and adjustment of pH to 9.0. PCD (100×) was made by the addition of 9.3 mg of PCD (PCD, number P8279, Sigma-Aldrich) to 13.3 ml of buffer (100 mM Tris-HCl, pH 8.0, 50 mM KCl, 1 mM EDTA, 50% glycerol).

### DNA-PAINT imaging buffer

1× PBS, 1 mM EDTA, 500 mM NaCl, pH 7.4, 0.02% Tween, optionally supplemented with 1× Trolox, 1× PCA and 1× PCD. Tween-20 (number P9416-50ML), protocatechuate 3,4-dioxygenase pseudomonas (PCD, number P8279), 3,4-dihydroxybenzoic acid (PCA, number 37580-25G-F), 1× PBS (pH 7.2, number 20012-019) and (±)-6-hydroxy-2,5,7,8-tetra-methylchromane-2-carboxylic acid (Trolox, number 238813-5G) were ordered from Sigma-Aldrich. EDTA (number AM9260G) and 1× PBS (pH 7.2, number 20012-019) were purchased from Thermo Fisher Scientific.

### Metabolic incorporation of Ac_4_GalNAz/Ac_4_ManNAz, click of docking strands and fixation

A few hours after seeding, the cells were supplemented with 50 μM of Ac_4_ManNAz (Thermo Fisher Scientific) or Ac_4_GalNAz (Thermo Fisher Scientific) in cell culture media. After 72 h of incubation, when cells had reached approximately 60% confluency, they were treated with 50 μM of each of the 6 docking DNA strands in culture media for 2 h at 37 °C to allow for the click reaction between the docking DNA sequences and the azido sugars. Following this incubation, the cells were fixed with 4% paraformaldehyde (Sigma-Aldrich) in DPBS (Life Technologies) for 20 min at room temperature. The cells were then permeabilized using 0.1% Triton X-100 (Thermo Fisher Scientific) in DPBS for 10 min at room temperature. To ensure thorough removal of unreacted reagents and minimize background, cells were washed three times with DPBS at each step, including before and after the click reaction, fixation and permeabilization procedures.

### Preparation of STORM samples

For STORM imaging, samples were prepared using the same protocol as described above, with one modification: instead of docking DNA strands, 50 μM of DBCO-647 (Jena Bioscience) was used for the click reaction.

### STORM imaging

A reducing oxygen scavenging buffer that induces blinking of single fluorophores was used according to the literature^55^. The STORM buffer consisted of 2 μl ml^−1^ catalase (Sigma-Aldrich, C100), 10% (w/v) glucose (BD Difco, 215530), 100 mM Tris-HCl (Thermo Fisher Scientific, 15567-027), 560 μg ml^−1^ glucose oxidase (Sigma-Aldrich, G2133) and 20 mM cysteamine (Sigma-Aldrich, 30070). The PBS in which the fixed cells were stored was replaced by the blinking buffer. First, diffraction-limited imaging was performed with low-intensity illumination of 1 W cm^−^^2^. Then, the laser power was increased to ~1.2 kW cm^−^^2^. Image acquisition was started after a short delay to ensure that most fluorophores were shelved into a dark state. The exposure time was 50 ms, and 40,000 frames were obtained for sialic acid imaging.

### RESI imaging

**Extended Data Fig. 6:**
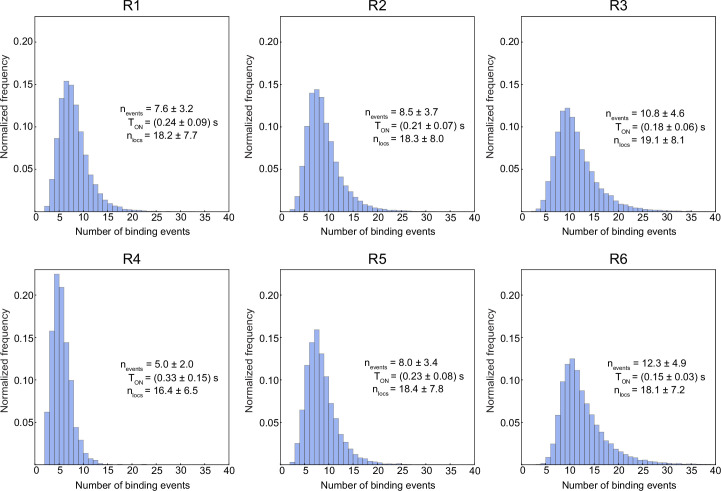
Binding kinetics of individual channels. Histograms of binding events of the clusters of individual channels R1 to R6. Values of mean and standard deviation of number of binding events (n_events_), binding time (T_ON_) and number of localizations (n_locs_) are displayed in the figure for each individual channel. The binding kinetics were obtained from an area of 85 μm^2^ of the ManNAz dataset. Source data

Gold nanoparticles (Cytodiagnostics, number G-90-100) were diluted 1:2 in PBS and incubated for 10 min at RT and the sample was washed two times with PBS to remove unbound gold. First, the imager solution (Extended Data Table 1) in DNA-PAINT imaging buffer for the first round was incubated for 2 min and then replaced with a fresh imager, after which the first acquisition round was started. The sample was washed with at least 2 ml of PBS between imaging rounds until no residual signal from the previous imager solution was detected. Then, the next imager solution was introduced. RESI imaging was conducted via six subsequent DNA-PAINT imaging rounds with only one of the imagers in each round. The number of imaging rounds was determined according to the calculations previously described (EDF14 in Reinhardt et al.^30^) such that the fraction of non-resolvable molecules within each round was <10% (average 6%). The optimal imager concentration required to achieve sparse blinking can vary. In this study, we utilized concentrations up to 125 pM. These concentrations were adjusted to ensure that blinking events were frequent enough yet sparse enough to maintain high DNA-PAINT resolution. The optimal concentration for each dataset was determined through visual assessment of the blinking and remained consistent across the corresponding set to allow for meaningful comparisons. The blinking kinetics were subsequently checked to ensure that the sampling was indeed sufficient as shown in Extended Data Fig. 6. In each FOV, 40,000 frames with 100 ms exposure time per frame were acquired. A laser power of 35 mW (560 nm laser, measured after the objective) was used, corresponding to a power density of ~175 W cm^−^^2^. Further details on imaging parameters are provided in Extended Data Table 1.

### Microscopy set-up

Fluorescence imaging was carried out on an inverted microscope (Nikon Instruments, Eclipse Ti2) with the Perfect Focus System, applying an objective-type TIRF configuration equipped with an oil-immersion objective (Nikon Instruments, Apo SR TIRF ×100, NA 1.49, Oil). A 560 nm laser (MPB Communications, 1 W) was used for excitation and coupled into the microscope via a Nikon manual TIRF module. The laser beam was passed through a cleanup filter (Chroma Technology, ZET561/10) and coupled into the microscope objective using a beam splitter (Chroma Technology, ZT561rdc). Fluorescence was spectrally filtered with an emission filter (Chroma Technology, ET600/50m and ET575lp) and imaged on an sCMOS camera (Hamamatsu Fusion BT) without further magnification, resulting in an effective pixel size of 130 nm after 2 × 2 binning. TIR illumination was used for all measurements. The central 1,152 × 1,152 pixels (576 × 576 after binning) of the camera were used as the region of interest. The scan mode of the camera was set to ‘ultra quiet scan’ (readout noise = 0.7 e^−^ r.m.s., 80 μs readout time per line). Raw microscopy data were acquired using μManager (version 2.0.1)^56^.

### Single-molecule localization analysis

Raw fluorescence data were reconstructed using the Picasso software package^57^ (the latest version is available at https://github.com/jungmannlab/picasso). Drift correction was performed using the AIM algorithm^58^ with gold nanoparticles as fiducials for all experiments. The six channels were aligned through cross-correlation of the fiducial gold nanoparticles using Picasso. DNA-PAINT images shown in Fig. 2 are obtained by merging all six RESI acquisition channels after alignment.

### RESI analysis

**Extended Data Fig. 7:**
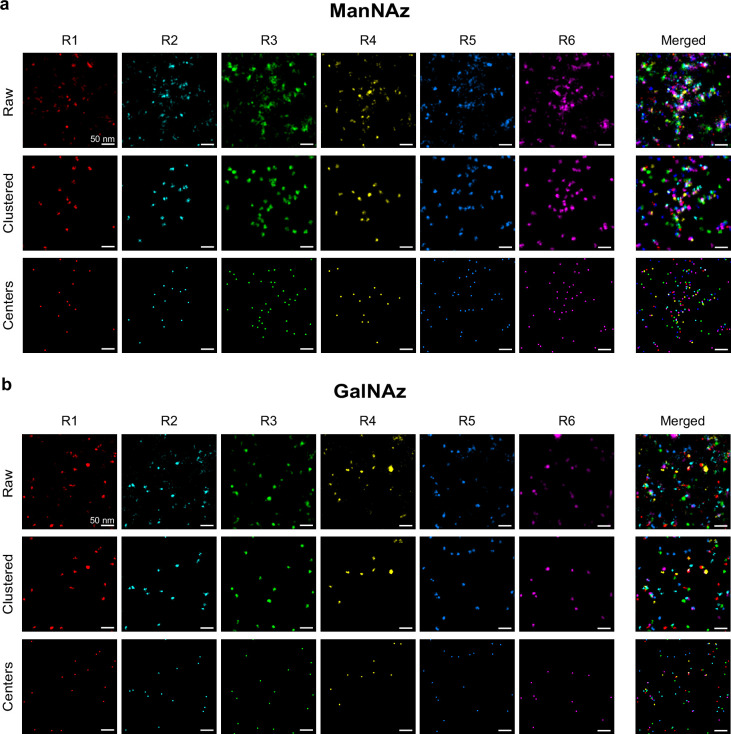
Step-by-step analysis of individual channel. **a**,**b**, Exemplary zoomed-in regions for ManNAz (**a**) and GalNAz (**b**), displaying the individual exchange rounds (R1 to R6). Raw localizations (“Raw”), clustered localizations (“Clustered”) and cluster centers (“Centers”) are displayed. A merged image of the channels (keeping their identity color-coded) is displayed on the right side of the figure (“Merged”). Scale bar: 50 nm.

RESI analysis was carried out as described previously^30^. In brief, the localizations in each of the six channels were clustered using the custom clustering algorithm described previously. The clustering algorithm uses two input parameters: radius *r*, which sets the final size of the clusters and defines a circular environment around each localization, and the minimal number of localizations, *n*_min_, representing a lower threshold for the number of DNA-PAINT localizations in any cluster. We used a radius *r* = 7.15 nm and *n*_min_ = 10 localizations. The radius value *r* is chosen to be approximately 2.35 times our localization precision, which yields optimal results in terms of false detections. The minimum number of *n*_min_ = 10 localizations ensures the distinction of repetitively sampled binding sites (single sugars) from unspecific binding events of the imagers. This number was determined by analysing the binding kinetics of the individual binding sites on each channel (R1 to R6 sequences). Extended Data Fig. 6 shows the binding kinetics and the statistics of each channel. The *n*_min_ parameter was chosen to ensure that sufficient sampling is achieved, and false positives are minimized, that is, the probability of imagers binding repeatedly for >10 locs in an unspecific area of radius *r* on the cell surface is very low, while the probability of detecting >10 locs for a binding site is high. Quantitatively, we chose *n*_min_ ≈ *n*_mean_ − *n*_std_ as a parameter that provides a good compromise of low false positives and low false negatives. We note that, in principle, fine-tuned parameters *r* and *n*_min_ can be set to a different value for each channel according to specific binding kinetics and localization precisions. We chose to use the same *r* and *n*_min_ for all channels since the localization precisions and localizations statistics were similar across the six channels in our experiments. To further minimize false positives, clusters of localizations are subsequently analysed in the time domain (time traces) and unspecific binding events are filtered out. Finally, the centre of each cluster is calculated and the cluster centres of all six channels are merged to produce the RESI image. Extended Data Fig. 7 shows the step-by-step analysis (raw localizations, clustered localizations, cluster centres) for the six individual channels (R1 to R6) for two exemplary zoom-ins, one for ManNAz and one for GalNAz.

### Quantitative analysis of RESI data

**Extended Data Fig. 8:**
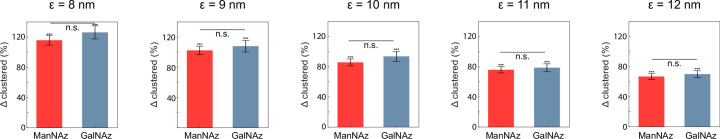
DBSCAN analysis with different ε parameters. Results of the DBSCAN analysis varying the ε parameter in the DBSCAN algorithm from ε = 8 nm to ε = 12 nm. Variation of the ε parameter changes the clusterization, but the trend is conserved: significant Δ Clustered (with respect to CSR) values are obtained in all cases, and non-significant differences between ManNAz and GalNAz are always retrieved. Height of the bar plot represents the mean value and error bar represents the standard error of the mean. Statistics derived from n = 40 different areas picked from 6 different cells (technical replicate) for ManNAz and n = 40 different areas picked from 6 different cells (technical replicate) for GalNAz. A one-sided t-student test was used to compare Δ clustered to the null hypothesis, a two-sided t-student test was used to compare Δ clustered for ManNAz and GalNAz. ‘***’ indicates p-value < 0.001 and ‘n.s.’ indicates p-value > 0.05. Exact p-values are the following: for ε = 8 nm; p-value^ManNAz^ = 2.9 × 10^−20^, p-value^GalNAz^ = 4.1 × 10^−17^, p-value^ManNAz - GalNAz^ = 0.34, for ε = 9 nm; p-value^ManNAz^ = 1.2 × 10^−20^, p-value^GalNAz^ = 9.3 × 10^−17^, p- value^ManNAz - GalNAz^ = 0.56, ε = 10 nm; p-value^ManNAz^ = 8.9 × 10^−21^, p-value^GalNAz^ = 4.3 × 10^−17^, p-value^ManNAz - GalNAz^ = 0.34, for ε = 11 nm; p-value^ManNAz^ = 1.7 × 10^−20^, p-value^GalNAz^ = 1.3 × 10^−17^, p-value^ManNAz - GalNAz^ = 0.69, for ε = 12 nm; p-value^ManNAz^ = 6.2 × 10^−20^, p-value^GalNAz^ = 6.4 × 10^−18^, p-value^ManNAz - GalNAz^ = 0.59. Source data

Downstream analysis (DBSCAN and NNDs) of the RESI data was performed using custom-written Python scripts based on Numpy^59^ functions and Scipy^60^ implementations of DBSCAN^37^ and KD-tree nearest-neighbour search^61^. The DBSCAN parameters were *ε* = 10 nm and minPts = 2. The number of areas analysed was *N* = 40 for both ManNAz and GalNAz. The areas were selected from *M* = 6 different cells for ManNAz and GalNAz respectively from two different FOVs for each sample. Extended Data Fig. 8 shows that varying the *ε* parameter from *ε* = 8 nm to *ε* = 12 nm changes the clusterization, but the trend is conserved: significant Δ clustered (with respect to CSR) values are obtained in all cases, and non-significant differences between ManNAz and GalNAz are always retrieved.

### Computational sugar–sugar distance analysis

To measure sugar–sugar distances within glycans, we performed structural modelling and analysis using Chimera^62^ version 1.18. Glycan conformations were optimized via Gasteiger energy minimization to ensure accurate modelling. Distances were then computationally determined by measuring the spatial separation between the carbon atoms nearest to the anchor points of the experimental labels. This approach provided theoretical distance estimations, aligning the computational model with the expected experimental labelling positions.

### Statistics and reproducibility

No statistical method was used to predetermine sample size. No data were excluded from the analyses. The experiments were not randomized. The investigators were not blinded to allocation during experiments and outcome assessment.

## Online content

Any methods, additional references, Nature Portfolio reporting summaries, source data, extended data, supplementary information, acknowledgements, peer review information; details of author contributions and competing interests; and statements of data and code availability are available at 10.1038/s41565-025-01966-5.

## Source data

Source Data Fig. 3 Statistical source data.

Source Data Extended Data Fig. 2 Statistical source data.

Source Data Extended Data Fig. 4 Statistical source data.

Source Data Extended Data Fig. 5 Statistical source data.

Source Data Extended Data Fig. 6 Statistical source data.

Source Data Extended Data Fig. 8 Statistical source data.

## Data Availability

Localization data are available via Zenodo at 10.5281/zenodo.14826252 (ref. ^63^). Raw microscopy data obtained during this study are available from the corresponding authors on reasonable request. Analysis code is available via GitHub at https://github.com/lumasullo/glycans-resi (ref. ^64^).

## Extended data

**Extended Data Table 1:** Acquisition parameters

Data	Round	Exposure (ms)	Frames	Laser power (at the sample, mW)	Imagers	Buffer	Cluster radius *r* (nm)	Cluster *n*_*n*_
**(d)STORM** Fig. 2 **(a-d)**	Round 1	50	40 000	200	Alexa 647	STORM buffer	-	-
**RESI** Fig. 2**(i-l)**, Fig. 3	Round 1	100	40 000	35	125 pM R1	DNA-PAINT buffer	7.15	10
	Round 2		40 000		50 pM R2			
	Round 3		40 000		50 pM R3			
	Round 4		40 000		5 pM R4			
	Round 5		40 000		125 pM R5			
	Round 6		40 000		125 pM R6			
Extended Data Fig. 1	Round 1	100	40 000	35	125 pM R3	DNA-PAINT buffer	-	-
Extended Data Fig. 2	Round 1	100	20 000	35	250 pM R1	DNA-PAINT buffer	-	-

Imaging acquisition parameters.

**Extended Data Table 2:** Docking and imager DNA sequences

Docking strand name	Sequence (5’ -> 3’)	5'mod
5xR1	TCCTCCTCCTCCTCCTCCT	DBCO
5xR2	ACCACCACCACCACCACCA	DBCO
7xR3	CTCTCTCTCTCTCTCTCTC	DBCO
7xR4	ACACACACACACACACACA	DBCO
5xR5	CTTCTTCTTCTTCTTCTTC	DBCO
5xR6	AACAACAACAACAACAACAA	DBCO
**Imager name**	**Sequence** **(5’ -> 3’)**	**3'-mod**
R1	AGGAGGA	Cy3b
R2	GGTGGT	Cy3b
R3	GAGAGAG	Cy3b
R4	TGTGTGT	Cy3b
R5	GAAGAAG	Cy3b
R6	TGTTGTT	Cy3b

DNA sequences of the docking strands and imagers that were used.

## Code availability

Custom analysis code is available via GitHub at https://github.com/lumasullo/glycans-resi (ref. ^64^).
